# Use of Neutrophil-to-Lymphocyte Ratio to Predict In-Hospital Mortality in Patients Admitted with Acute Decompensation of Atrial Fibrillation

**DOI:** 10.3390/jcm13164719

**Published:** 2024-08-12

**Authors:** Nilima Rajpal Kundnani, Abhinav Sharma, Daniel Florin Lighezan, Doina Georgescu, Stelian I. Morariu, Daniel Dumitru Nisulescu, Romina Georgiana Bita, Ciprian Ilie Rosca

**Affiliations:** 1 Discipline of Internal Medicine and Ambulatory Care, Prevention and Cardiovascular Recovery, Department VI—Cardiology, “Victor Babeș” University of Medicine and Pharmacy, 3000041 Timișoara, Romania; knilima@umft.ro (N.R.K.); sharma.abhinav@umft.ro (A.S.); 2 Research Centre of Timisoara Institute of Cardiovascular Diseases, “Victor Babeș” University of Medicine and Pharmacy, 3000041 Timișoara, Romania; 3 Department V, Internal Medicine I—Discipline of Medical Semiology I, Center of Advanced Research in Cardiology and Hemostaseology, “Victor Babeș” University of Medicine and Pharmacy, Eftimie Murgu Sq. no. 2, 300041 Timișoara, Romania; dlighezan@umft.ro (D.F.L.); rosca.ciprian@umft.ro (C.I.R.); 4 General Medicine Faculty, “Vasile Goldiș” West University Arad, 473223 Arad, Romania; daniel.nisulescu@umft.ro; 5 General Medicine Faculty, “Victor Babeș” University of Medicine and Pharmacy, Eftimie Murgu Sq. no. 2, 300041 Timișoara, Romania; 6 2nd Department, Radiology and Medical Imaging, General and Dento-Maxillary Imaging, Dental Medicine Faculty, “Victor Babeș” University of Medicine and Pharmacy, 3000041 Timișoara, Romania; romina.bita@umft.ro

**Keywords:** neutrophil-to-lymphocyte ratio (NLR), in-hospital mortality, atrial fibrillation (AF), CHA_2_DS_2_-VASc score, HAS-BLED score

## Abstract

**Background/Objectives:** The prevalence of atrial fibrillation (AF) has been on the rise over the last 20 years. It is considered to be the most common cardiac arrhythmia and is associated with significant morbidity and mortality. The need for in-hospital management of patients having AF is increasing. Acute decompensation of cardiac rhythm is an indication for hospital admission. In the existing literature, several studies on different pathologies have observed that the risk of death was greater for patients with an increased neutrophil-to-lymphocyte ratio (NLR) and suggested that the NLR can be a useful biomarker to predict in-hospital mortality. This study aims to evaluate the link between the neutrophil-to-lymphocyte ratio at admission and death among the patients admitted to the medical ward for the acute manifestation of AF, and to gain a better understanding of how we can predict in-hospital all-cause death based on the NLR for these patients. **Methods:** A single-center retrospective study in an academic medical clinic was conducted. We analyzed if the NLR at in-hospital admission can be related to in-hospital mortality among the patients admitted for AF at the Medical Ward of Municipal Emergency University Hospital Timisoara between 2015 and 2016. After identifying a total of 1111 patients, we divided them into two groups: in-hospital death patients and surviving patients. We analyzed the NLR in both groups to determine if it is related to in-hospital mortality or not. One patient was excluded because of missing data. **Results:** Our analysis showed that patients who died during in-hospital admission had a significantly higher NLR compared to those who survived (*p* < 0.0001, 95% CI (1.54 to 3.48)). The NLR was found to be an independent predictor of in-hospital death among patients with AF, even for the patients with no raised level of blood leukocytes (*p* < 0.0001, 95% CI (0.6174 to 3.0440)). Additionally, there was a significant correlation between the NLR and the risk of in-hospital death for patients admitted with decompensated AF (*p* < 0.0001), with an area under the ROC curve of 0.745. Other factors can increase the risk of death for these patients (such as the personal history of stroke, HAS-BLED score, and age). **Conclusions:** The NLR is a useful biomarker to predict in-hospital mortality in patients with AF and can predict the risk of death with a sensitivity of 72.8% and a specificity of 70.4%. Further studies are needed to determine the clinical utility of the NLR in risk stratification and management of patients with AF.

## 1. Background

Atrial fibrillation (AF) is the most common cardiac arrhythmia [1,2], and its prevalence has been on the rise in the last 20 years [1,2,3]. The presence of AF is a risk factor per se when it is present in patients with subsequent comorbidities, and is related to an increased mortality rate regardless of underlying comorbidities [4]. Several risk scores for stroke-related AF have been developed, but the risk of death seems to have been somewhat neglected.

Any tool that can predict mortality in AF in-hospital patients will be very important for clinicians. Until a mortality prediction score is established, alarming signs can help clinicians to make better decisions for patients [5,6].

The correlation between death and stroke risk estimation scales is presented in some studies, but this research demonstrates no or a poor relationship between mortality due to AF and the value of the CHA_2_DS_2_-VASc (Congestive heart failure, Hypertension, Age ≥ 75 years [doubled], Diabetes, previous Stroke or transient ischemic attack [doubled], Vascular disease, Age 65–75 years, Sex) score [7,8,9]. In these studies, some correlation between the risk of death and atrial fibrillation occurrence in critical care units was made, but all from the point of view of other intermediate situations such as sepsis, stroke, and surgical or interventional procedures [6,10,11,12].

Several studies evaluated the association between different inflammatory markers and AF, and more recently, between those markers and AF-related death [13,14]. The increase in NLR values, induced by major psychological stress, can also be associated with an increase in the incidence of AF [15]. Increased values of myocardial necrosis markers in AF patients are associated with the risk of death, as evaluated with the CHA_2_DS_2_-VASc score [16]. Raised blood concentration of the coagulation biomarkers (such as D-dimer, thrombin antithrombin complex, prothrombin fragment 1 + 2, or soluble fibrin monomer complex) was associated with mortality [16]. Several studies have evaluated the association between various hematological indices and the risk of developing AF [17].

Our study aimed to evaluate the relationship between the neutrophil-to-lymphocyte ratio (NLR) at in-hospital admission and death among the patients admitted to the medical ward for the acute manifestation of atrial fibrillation in order to gain a better understanding of how we can predict in-hospital all-cause deaths.

## 2. Material and Methods

This retrospective study was conducted in the 1st Medical Ward at Municipal Emergency Hospital Timisoara. We analyzed patients admitted with AF between 1 January 2015 and 31 December 2016. Before starting this study, we obtained approval from the hospital management body and the Ethics Committee of the “Victor Babes” University of Medicine and Pharmacy, Timisoara.

**Figure 1:**
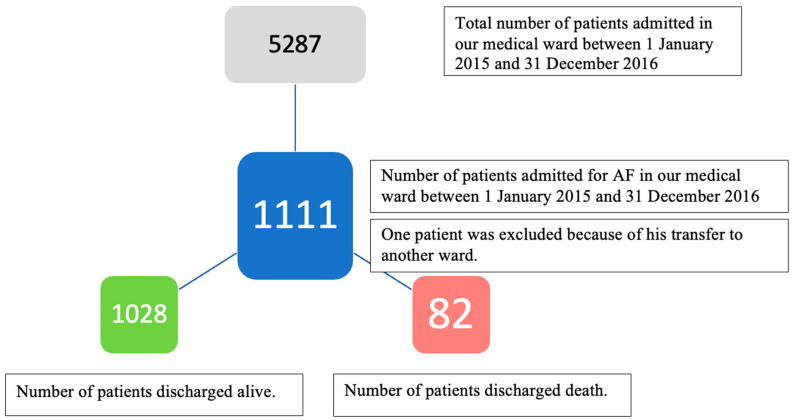
Flowchart of patient inclusion. We considered only the last admission if the patient had multiple admissions in that period. One additional patient was excluded from our analysis due to his transfer to the Neurology ward at another hospital and the lack of his discharged status (death or alive) from that department.

A total of 1111 cases with atrial fibrillation were identified. Patients were admitted to our hospital for an acute decompensation of atrial fibrillation, consisting of a very rapid ventricular response, regardless of the type of atrial fibrillation. For this research, patients were analyzed at the same place irrespective of the type of atrial fibrillation presented (paroxysmal, persistent, or permanent). However, for previous research related to the presence of neuropsychological complications, patients were divided into patients with paroxysmal atrial fibrillation respectively permanent, and patients with persistent AF were included in the group of patients with permanent AF. After patient identification, we divided these patients into two groups. The first group comprised surviving patients (1028 patients), and the second group comprised patients suffering in-hospital death (82 patients). Inclusion criteria consisted of diagnosis of AF present at in-hospital admission, regardless of AF type (paroxysmal or permanent) and mortality status. In the case of recurrent or multiple admissions, only the latest admission was included for data analysis (Figure 1). The exclusion criterion for this study was the absence of AF. We did not exclude patients according to their age, gender, religious or political beliefs, number of previous hospitalizations related to AF evolution, or associated comorbidities or treatments previous to the current hospital admission. One patient was excluded from our analysis due to his transfer to the Neurology ward in another hospital and the lack of information regarding his mortality status.

We calculated the NLR using numeric values obtained from the automatic blood counting apparatus at the time of admission to the hospital. Thereafter, we correlated its values with the occurrence of mortality and other factors that can increase the odds of the in-hospital mortality of our patients.

Overweight and obesity were stratified according to body mass index (BMI). Metabolic syndrome was established according to the International Diabetes Federation [18]. The glomerular filtration rate (GFR) was calculated using the 2012 CKD-EPI formula, and chronic kidney disease (CKD) was staged according to the KDOQI guidelines [19]. Hypertension was graded according to the 2018 Guideline for the Management of Hypertension endorsed by the European Society of Cardiology and the European Society of Hypertension. For heart failure (HF) classification, we used the planimetric ejection fraction (EF) and clinical staging according to the New York Heart Association (NYHA). Pulmonary hypertension (PHT) was estimated using tricuspid regurgitation and peak systolic artery pressure (PSAP) and classified as mild for PSAP between 30 and 44 mmHg, moderate between 45 and 70 mmHg, and severe for PSAP over 70 mmHg.

Statistical analysis was performed using MedCalc version 20.0 software with a significant *p*-value of *p* < 0.05. We use descriptive statistics, figures, and tables to summarize our findings. Results for targeted variables are presented using descriptive statistics (mean, standard deviation, range, median, and associated interquartile range) for continuous data, and counts with associated percentages for categorical data. Quartiles were obtained with Tukey’s method. An independent samples *t*-test was used to analyze the differences in means for continuous variables, while differences between categorical variables were examined by the Chi-squared test or with Fisher’s exact test. Categorical data are presented as counts (percentages). Logistic regression analysis was used to evaluate the odds of mortality.

Ethics approval: Written informed consent was obtained from all patients, as a part of a routine procedure at the time of admission, who agreed their data can be used for research purposes while keeping their identity and personal information confidential. Furthermore, ethics approval was obtained from the Ethics Committee board of the Hospital where the study was performed (E-1991/2024).

## 3. Results

Our study groups were comparable from a gender point of view. In terms of age, we found that the patients enrolled in group 1 (survivors) had a younger mean and median age (the youngest patient was 26 years old, and the oldest patient was 97 years old) as compared with group 2 patients (who died), where the youngest patient was 46 years old, and the oldest patient was 94 years old (Table 1).

It was concluded that younger patients were more likely to survive during hospitalization for an acute episode of atrial fibrillation (*p* < 0.001).

For the CHA_2_DS_2_-VASc score and HAS-BLED score, their mean values were higher in the group of deceased patients, with a statistical significance (*p* < 0.0001) for the second group (Table 2). Hence, we concluded that these scores can be used in providing an estimation of the risk of death (predicting in-hospital mortality) among the patients admitted for acute decompensation or the first appearance of AF.

The comorbidities accompanying patients with atrial fibrillation can play an important role in the survival of patients admitted for acute manifestations of atrial fibrillation. Among several comorbidities as listed in Table 3, the incidence of most has shown statistical significance (Table 3). We observed that obesity, metabolic syndrome, arterial hypertension, and pulmonary hypertension are significantly present among patients who have survived, while in patients with a history of stroke, mortality rates were significantly higher. The prevalence of all listed comorbidities, except a history of stroke, is paradoxically on the lower side in the group of patients having a mortality outcome. Interestingly, a history of stroke is more prevalent in the group of patients having mortality. It can be argued that having a stroke increased the odds of mortality; however, further studies are required. Other comorbidities do not seem to contribute to mortality in patients admitted with AF.

The paraclinical investigations that influenced mortality in our study are mentioned in Table 4. 

Elevated average values of LVEF, ESR, LDL cholesterol, GFR, as well as serum uric acid were found in group 1 patients with acutely decompensated AF or its first manifestation. They may have a role in reducing mortality as such. On the other hand, elevated average values of the neutrophil count and the neutrophil/lymphocyte ratio were associated with an increased incidence of death (*p*-value recorded in both situations being *p* < 0.0001). 

Even though the average values of the leukocyte count were not quantified for each patient to simplify it as leukocytosis being “present or absent”, the presence of leukocytosis was significantly associated with a higher death rate (57.3% of deceased patients had leukocytosis, compared to 27.4% recorded among patients who survived hospitalization, *p* < 0.0001).

To see whether the presence of leukocytosis influences the predictive role of the neutrophil/lymphocyte ratio, we extracted the patients without leukocytosis from both groups and re-analyzed the behavior of the neutrophil/lymphocyte ratio among the remaining patients (Table 5). In this situation, we found that the deceased patients throughout the hospitalization had higher average values of the neutrophil/lymphocyte ratio, with this difference also presenting statistical significance (*p* = 0.0041)

The impact on the risk of death imposed by these factors was assessed using logistic regression via the Enter method and the Chi-square test, and the obtained data are presented in Table 6.

Equipped with these data, we questioned what would be the limit at which the neutrophil/lymphocyte ratio could be used to distinguish patients at high risk of death. 

To be able to answer this question, we divided the value of the neutrophil to lymphocyte ratio into quartiles for the CHA2DS2-VASc score and the HAS-BLED score of the patients in both groups (divided automatically by the software for statistical processing).

The results of quartiles of the neutrophil/lymphocyte ratio are presented in Table 7 and Table 8.

Regarding in-hospital mortality, it was recorded both among patients with paroxysmal AF and patients with permanent AF (Table 9), and the number of deaths recorded among patients with permanent AF was significantly higher.

Applying the ANOVA test to these quartiles, it was found that there is a statistical difference between each quartile in terms of the neutrophil/lymphocyte ratio value and the number of deaths. Also, an increase in the value of the ratio above 3.644 is associated with a statistically significant increase in the mortality rate, with patients in quartiles 3 and 4 dying much more frequently (*p* < 0.0001). 

The quartiles of the CHA2DS2-VASc score are presented in Table 10 and Table 11.

The death of patients is much more frequent in the q4—CHA2DS2-VASc score, with a statistically significant difference (Chi-square test, *p* < 0.0001).

In the case of the NLR, by applying the ANOVA test, we demonstrated that there is a statistically significant difference among the four quartiles of the CHA2DS2-VASc score mean values, as well as that there are statistical differences among the mortality rates of the patients included in these quartiles (*p* < 0.0001). Like the NLR frame, there are statistically significant differences between mortality and the NLR value within the quartiles (*p* < 0.0001).

The value of the NLR correlates with the value of the CHA2DS2-VASc score, with high values of the CHA2DS2-VASc score being associated with increased values of the NLR, but with a higher average value of the CHA2DS2-VASc score within the q3-NLR (Table 12).

Regarding the HAS-BLED score, the discussion can be bi-directional. The increase in the HAS-BLED score value is associated with the increase in the rate of death, with statistical significance (*p* < 0.0001). Despite this association, the increase in the HAS-BLED score is not significantly associated with the increase in the NLR value, even if an increased HAS-BLED score associates with higher values of the NLR for the quartiles in which it was analyzed. 

**Figure 2:**
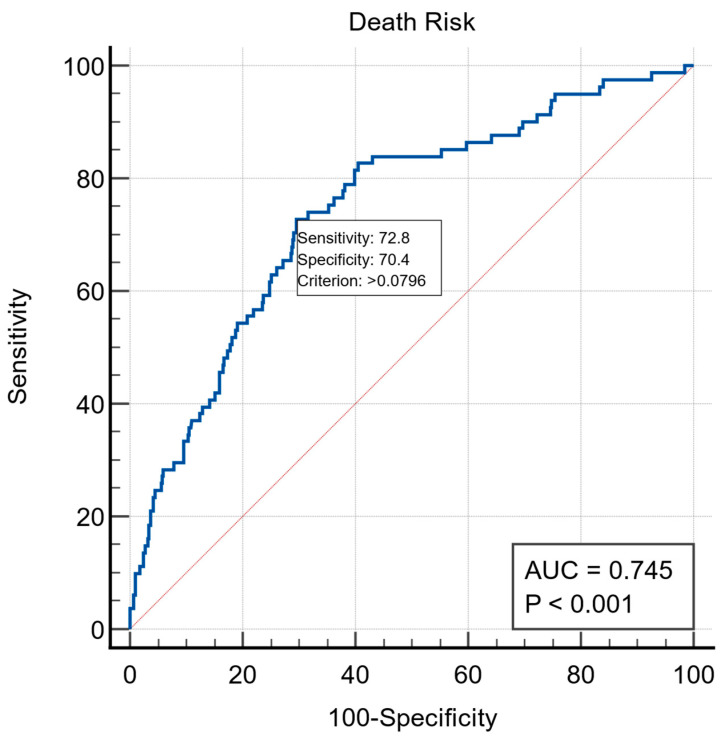
Area under the ROC curve for the estimation of risk of mortality using the NLR.

In our study, the risk of mortality using the NLR (Figure 2) was estimated using the area under the ROC curve (AUC), and it could be quantified with a specificity of 70.4% and a sensitivity of 72.8% (*p <* 0.0001 for Area = 0.5; 95% CI [0.718 to 0.770]; standard error = 0.0289 and z statistic = 8.479). The formula used is (−7.77619 + 0.69170 * Stroke + 0.32134 * HAS-BLED + 0.039662 * Age + 0.083524 * NLR), where Stroke is 1 if it is present and 0 (zero) if there is no history of stroke, HAS-BLED is the value of the HAS-BLED score, age is the patient’s age in years, and NLR is the value of the neutrophil/lymphocyte ratio.

## 4. Discussion

A score that can predict mortality in AF in-hospital patients will be a very important tool for any clinician, both for the proper management of the patient’s disease and for appropriate family information about the prognostic outcomes and short-term evolution. Inflammation and its markers appear to play an important role in predicting death or cardio-embolic complications in patients with AF. Ma et al. demonstrated the association of all-cause mortality with IL-34 and IL-38 in patients with AF [13]; this association is also demonstrated for other ILs (1, 6, 16) [20].

Our study found that the NLR at the time of hospitalization turns out to be a very useful tool to predict the mortality of a patient having AF, irrespective of other comorbidities, ongoing treatments, or other variables. Since almost all patients being hospitalized would undergo a complete blood count (hemogram) report, the NLR is an inexpensive and handy tool. Moreover, it is simple to use since it does not involve any complicated parameters or variables that could require any of patients’ other pre-existing data or information.

In this study, we evaluated the association of the NLR with mortality, which was found to be an independent predictor of mortality in patients admitted for acute decompensation or the first manifestation of AF. After that, because of the findings, we wanted to explore the association of the CHA_2_DS_2_-VASc score, NLR, and mortality, and to determine if this association is also an independent predictor of death.

The values of the NLR in patients with AF are higher in comparison with those without AF [21]. An analysis from the ENGAGE AF-TIMI 48 trial made by Fagundes et al. [22] indicates an increase in death (among other complications) in those patients with an increase in the NLR. The surgery for aortic valve replacement was also associated with an increase in death among the patients with an increase in the NLR in patients with AF. They demonstrated that an NLR value over 1.5 is associated with an increased incidence of mortality. In our study, values exceeding 3.4 were associated with an increased incidence of mortality. 

Furthermore, increased values of the NLR were associated with higher mortality in patients without AF after elective off-pump coronary artery bypass grafting [23].

ICU patients admitted for community-acquired pneumonia were also reported to have a higher short-term mortality rate among those with higher values of NRL, with a more accurate prediction rate when compared with the APACHE II score [23,24]. 

Our study confirms the previous data but with some particularities. First, even when we excluded patients with leukocytosis from the statistical analysis, the prognostic value was the same for the patients with higher values of the NLR. Second, our patients were admitted to a medical ward without the need for interventional or surgical maneuvers that can increase the inflammatory status via increasing psychological stress or by the intervention itself.

In our analysis of the risk of mortality, we used only the variables that were directly correlated with mortality and the NLR, such as the CHA_2_DS_2_-VASc score, HAS-BLED score, ESR, age, and stroke. The final formula retained only the presence of a history of past or recent stroke, the HAS-BLED score, age, and NLR because the other two parameters seem to adjust each other. On the other hand, obesity, metabolic syndrome, LDL cholesterol, arterial hypertension, and pulmonary hypertension were associated with a decreased risk of death because higher values of those parameters were found among the survival group of patients. It is paradoxical since such comorbidities cannot have any sort of protective effect from mortality. These findings are in part discussed in our previous work regarding the incidence of neuro-psychic complications of AF and the effect of metabolic syndrome on the risk of developing stroke in AF patients.

The underlying mechanisms responsible for an increase in mortality in patients with higher values of the NLR are not yet completely understood, but a more reliable explanation is that the increase in the NLR is associated with an excessive increase in the production of IL [25,26,27].

In the examined group, diabetes mellitus (DM) does not seem to influence the mortality rate nor the survival rate. Even the percentage of patients who presented with this condition is practically equal in both groups. This feature contrasts with the observation showing that the association of an increased systemic inflammatory index with the presence of DM constitutes a poor prognostic element of AF [28]. Further studies may help in either supporting or dismissing this observation.

The correlation between the mean value of the NLR and the mean value of the q-CHA2DS2-VASc score in patients who died during hospitalization could not be achieved due to the small number of deaths recorded in the analyzed group. However, starting from the result of the correlation performed for patients in Group 1, as well as based on the distribution of the average values of the NLR within the CHA2DS2-VASc score quartiles among the deceased patients, it can be stated that an increased average value of the two parameters is associated with increased mortality among patients hospitalized for acute decompensated or new-onset AF.

Numerous studies published recently have shown the effectiveness of predicting the risk of mortality of the CHA2DS2-VASc score for several categories of patients and various pathologies associated or not with AF, among which those who underwent PCI for STEMI are the most numerous. Increased all-cause mortality was associated with increased values of the CHA2DS2-VASc score, since the value of the CHA2DS2-VASc score of at least 5 is associated with a 3-fold increase in the death rate compared to patients with a CHA2DS2-VASc score with values less than 5 [29,30].

However, the association of the 3rd quartile of the NLR with higher values of the CHA2DS2-VASc score is interesting. An aspect observed with regard to the incidence of stroke in patients with AF is described in more detail in a previous work [31], and it is also acknowledged by other authors [32]. From this observation, it seems that a higher risk for a poor outcome among patients with AF (whether it is the incidence of stroke or that of mortality) is seen in patients with a CHA2DS2-VASc score having values of 5–6. Hence, it becomes more crucial for us to redefine the CHA2DS2-VASc score and to establish a more powerful tool [33,34,35,36,37]. 

In previous studies, the ENGAGE AF-TIMI 48 trial [22], the high values of the NLR were associated with the increased risk of acute bleeding episodes. Our study being retrospective, we were not able to analyze the presence of bleeding at the time of inclusion of patients. However, we can mention that none of the admitted patients had acute bleeding of any etiology at the time of admission to our hospital. In addition, in our hospital, patients with upper or lower digestive bleeding with hemodynamic impact are hospitalized in the Gastroenterology department, and those with hemorrhagic vascular accidents are referred for admission to the Neurology department. On the other hand, the bleeding that occurred before the presentation for hospitalization in the patients in our study group was obtained through anamnesis (history taking and previous medical records) and was mentioned in other papers [38]. Thus, seven hemorrhagic cerebrovascular accidents were registered, four male and three female patients, and of these, three patients were on antiplatelet treatment at the time of the hemorrhagic stroke, and four patients were on anticoagulant treatment with anti-vitamin K anticoagulants. The comorbidities of these patients were CKD stage 2 (two patients), stage 3a (two patients), stage 3b (one patient), and stage 5 (one patient); chronic coronary syndrome was present in three male patients and peripheral arterial disease in one male patient. The difference between the two recorded aspects (an increase in the risk of bleeding in one and the lack of bleeding in the other) can be attributed to the populations studied, but also to the anticoagulant treatment used, considering the fact that none of the patients followed in our study had received edoxaban (as this medication was not available in Romania at that time). Moreover, in the groups analyzed by us, we saw that the risk of bleeding assessed with the HAS-BLED score is average (Table 2), a situation in which, according to our experience, hemorrhagic events are usually absent.

There are also studies that describe the association between increased NLR values and left atrial appendage thrombosis or the presence of spontaneous echo-contrast in the left atrium in patients with AF [39]. In our study, within echocardiographic transthoracic examinations performed on patients showed no presence of spontaneous echo-contrast nor of intra-atrial or intra-ventricular thrombi nor of left appendage thrombosis. 

Moreover, there are studies that associate the incidence or extent of stroke with the NLR value [22,40]. In the group of patients analyzed, none had a stroke during their hospital stay, nor did they indicate the presence of an acute stroke as the reason for presenting to the hospital.

Addition of new elements to the CHA2DS2-VASc score may increase the accuracy of predicting all-cause mortality among patients with AF [13].

Limitations of the study: One limitation of this study is the relatively small amount of data for cases that suffered mortality. Moreover, the fact that our study was retrospective can be considered as a major limitation of the study. Our study was conducted in a single center, which is a limitation. Even though we evaluated the relationship between the NLR and death in patients without leukocytosis, we were not able to evaluate other acute inflammatory markers. The lack of dosage of biomarkers is another limitation, which in future studies we plan to overcome, as it can provide us with better insight. In addition, the lack of clear cut-offs of the analyzed ratio or the NLR normograms led us to use the average NLR values as the cut-off, which can be considered another limitation of studies based on this aspect. 

## 5. Conclusions

The NLR can be a handy, simple, easy, and inexpensive tool to predict in-hospital mortality for patients admitted with new-onset or acute decompensation of AF. The association of new elements with the CHA2DS2-VASc score can increase its accuracy in predicting cardio-embolic events and death among patients with AF. For this, it is necessary to carry out extensive, multifactorial studies, which in the end can confer additional items to the CHA2DS2-VASc score for its better clinical utility.

## Figures and Tables

**Table 1:** Age and gender distribution of patients in the two groups.

	Group 1 *n* = 1028	Group 2 *n* = 82	*p*-Value
Age (mean)—years	71.90	77.58	*p* < 0.0001 (95% CI: 3.3222 to 8.0431)
Age (median)—years	73 (26–97)	80 (46–94)	*p* < 0.0001
Gender	F = 52%	F = 53.7%	*p* = 0.7719

*n*—number of patients; F—female gender.

**Table 2:** Mean of CHA_2_DS_2_-VASc and HAS-BLED scores for group 1 and group 2 patients.

	Group 1 *n* = 1028	Group 2 *n* = 82	*p*-Value
CHA_2_DS_2_-VASc	4.92 (95% CI: 4.80 to 5.03)	5.86 (95% CI: 5.45 to 6.27)	*p* < 0.0001 (95% CI: 0.51 to 1.36)
HAS-BLED	4.10 (95% CI: 4.02 to 4.17)	4.75 (95% CI: 4.48 to 5.02)	*p* < 0.0001 (95% CI: 0.38 to 0.92)

HAS-BLED—Hypertension, Abnormal liver function, abnormal renal function, Stroke, Bleeding tendency or predisposition, Labile INRs in patients under warfarin treatment, Elderly (age > 65 years), Drugs (antiplatelet agents, non-steroidal anti-inflammatories, alcohol abuse); CHA_2_DS_2_-VASc—Congestive heart failure, Hypertension, Age ≥75 years [doubled], Diabetes, previous Stroke or transient ischemic attack [doubled], Vascular disease, Age 65–75 years, Sex.

**Table 3:** Comparison of comorbidities and treatment with an increased influence on the risk of mortality in both groups. Characterization of the groups in terms of comorbidities and treatment with an impact on mortality.

	Group 1 *n* = 1028	Group 2 *n* = 82	*p*-Value
Obesity	26.8%	12.2%	*p* = 0.0037
Metabolic syndrome	24.9%	13.4%	*p* = 0.0192
High blood pressure	85.8%	61%	*p* < 0.0001
Diabetes mellitus	30.4%	30.5%	*p* = 0.9792
Stroke	39.2%	63.4%	*p* < 0.0001
Pulmonary hypertension	44.7%	25.6%	*p* = 0.0008
Dyslipidemia	40.0%	50.0%	*p* = 0.1200
Chronic obstructive pulmonary disease	20.0%	22.0%	*p* = 0.6478
Antiarrhythmic medications	30.0%	35.0%	*p* = 0.5373
Antidiabetic medications	20.0%	25.0%	*p* = 0.7771
Beta blockers	60.0%	65.0%	*p* = 0.4663
Statins	45.0%	70.7%	*p* = 0.0011
ACE inhibitors	50.0%	55.0%	*p* = 0.4639
Angiotensin receptor blockers—sartans	35.0%	30.0%	*p* = 0.3892
Diuretics	40.0%	45.0%	*p* = 0.4425
Spironolactone	25.0%	20.0%	*p* = 0.4196
Aspirin	30.0%	25.0%	*p* = 0.8231

*n*—number of patients.

**Table 4:** The paraclinical investigations with an impact on the mortality of patients with atrial fibrillation.

	Group 1 *n* = 1028	Group 2 *n* = 82	*p*-Value
LVEF	50.62% (49.20 to 52.04)	46.88% (35.49 to 58.28)	*p* = 0.3937 95% CI (12.34 to 4.87)
ESR	25.2442 (23.61 to 26.87)	17.439 (11.77 to 23.10)	*p* = 0.0105 95% CI (−13.78 to −1.82)
LDL cholesterol	91.2879 (88.60 to 93.96)	39.6951 (30.50 to 48.88)	*p* < 0.0001 95% CI (61.42 to −41.75)
GFR	39.38 (37.43 to 41.33)	27.78 (23.26 to 32.93)	*p* = 0.0012 95% CI (−18.62 to −4.58)
Uric acid	4.59 (4.36 to 4.82)	2.96 (2.04 to 3.89)	*p* = 0.0002 95% CI (−2.47 to −0.73)
Neutrophils	6.72 (6.42 to 7.02)	9.51 (8.45 to 10.57)	*p* < 0.0001 95% CI (1.69 to 3.89)
Neutrophil/lymphocyte ratio	4.67 (4.42 to 4.92)	7.18 (5.78 to 8.58)	*p* < 0.0001 95% CI (1.54 to 3.48)

LVEF—left ventricle ejection fraction; ESR—erythrocyte sedimentation ratio; GFR—glomerular filtration rate; CI—confidence interval.

**Table 5:** The expression of neutrophil/lymphocyte ratio in patients without leukocytosis.

	Group 1 without Leukocytosis (*n* = 738)	Group 2 without Leukocytosis (*n* = 37)	*p*-Value
Neutrophil/lymphocyte ratio (mean value)	3.7710 95% CI (3.5772 to 3.9649)	5.6017 95% CI (4.4028 to 6.8006)	*p* = 0.0041 95% CI (0.6174 to 3.0440)

*n*—number of patients; CI—confidence interval.

**Table 6:** The in-hospital risk of death for patients admitted with acute decompensation or a first episode of atrial fibrillation.

	Odds Ratio	*p*-Value	95% CI
Obesity	0.3803	*p* = 0.0050	0.1935 to 0.7475
Metabolic syndrome	0.4672	*p* = 0.0219	0.2437 to 0.8955
HBP	0.2586	*p* < 0.0001	0.1605 to 0.4167
Stroke	2.6839	*p* < 0.0001	1.6833 to 4.2792
PHT	0.4251	*p* = 0.0010	0.2550 to 0.7085
HAS-BLED score *	1.4422 *	*p* = 0.0045	1.1199 to 1.8572
CHA_2_DS_2_-VASc score *	1.1760 *	*p* = 0.0419	1.0060 to 1.3749
Leukocytosis	3.5524	*p* < 0.0001	2.2458 to 5.6191
Age quartile *	1.7117	*p* < 0.0001	1.3733 to 2.1335
Age *	1.0616 *	*p* < 0.0001	1.0350 to 1.0888
Neutrophil/Lymphocyte ratio Quartile *	1.6578 *	*p <* 0.0001	1.3278 to 2.0698
Neutrophil/Lymphocyte ratio *	1.0906 *	*p* < 0.0001	1.0512 to 1.1315
LDL cholesterol	0.0759	*p* < 0.0001	0.0454 to 0.1270

HBP—high blood pressure; PHT—pulmonary hypertension; HAS-BLED—Hypertension, Abnormal liver function, abnormal renal function, Stroke, Bleeding tendency or predisposition, Labile INRs in patients under warfarin treatment, Elderly (age > 65 years), Drugs (antiplatelet agents, non-steroidal anti-inflammatories, alcohol abuse); CHA_2_DS_2_-VASc—Congestive heart failure, Hypertension, Age ≥75 years [doubled], Diabetes, previous Stroke or transient ischemic attack [doubled], Vascular disease, Age 65–75 years, Sex; CI—confidence interval, * statistically significant parameter.

**Table 7:** The quartiles used in the statistical processing within the patients from group 1 (survivors).

	Neutrophil/Lymphocyte Ratio			
q-ratio	1	2	3	4
N	258	266	253	232
Minimum	0.0366	2.286	3.539	6.176
Maximum	2.285	3.536	6.143	34.826
Mean	1.581	2.851	4.544	10.336
95% CI	1.514 to 1.648	2.809 to 2.892	4.455 to 4.633	9.692 to 10.980

q-ratio—quartile neutrophil/lymphocyte ratio; N—number of patients; CI—confidence interval.

**Table 8:** The quartiles used in the statistical processing within the patients from group 2 (mortality).

	Neutrophil/Lymphocyte Ratio			
q-ratio	1	2	3	4
N	15	6	20	40
Minimum	0.105	2.406	3.644	6.180
Maximum	2.157	3.529	6.167	38.875
Mean	1.321	3.060	4.764	11.212
95% CI	0.997 to 1.644	2.540 to 3.579	4.479 to 5.049	9.023 to 13.400

q-ratio—quartile neutrophil/lymphocyte ratio; N—number of patients; CI—confidence interval.

**Table 9:** Mortality based on type of Atrial Fibrillation.

	Paroxysmal AF (*n* = 388)	Permanent AF (*n* = 722)	*p*-Values
Mortality	2.6% (*n* = 10)	10% (*n* = 72)	*p* < 0.0001
Neutrophil/lymphocyte ratio	4.2910 (95% CI 3.85 to 4.72)	4.8934 (95% CI 4.58 to 5.19)	*p* = 0.0236 (95% CI 0.08 to 1.12)

**Table 10:** The quartiles used in the statistical analysis of patients from group 1 (survival).

	CHA_2_DS_2_-VASc Score			
q-CHA_2_DS_2_-VASc	1	2	3	4
N	390	224	225	189
Minimum	0.000	5.000	6.000	7.000
Maximum	4.000	5.000	6.000	9.000
Mean	2.974	5.000	6.000	7.571
95% CI	2.864 to 3.085	5.000 to 5.000	6.000 to 6.000	7.481 to 7.662

q-CHA_2_DS_2_-VASc—quartile CHA_2_DS_2_-VASc score; N—number of patients; CI—confidence interval.

**Table 11:** The quartiles used in the statistical analysis of patients from group 2.

	CHA_2_DS_2_-VASc Score			
q-CHA_2_DS_2_-VASc	1	2	3	4
N	19	10	18	35
Minimum	1.000	5.000	6.000	7.000
Maximum	4.000	5.000	6.000	9.000
Mean	3.105	5.000	6.000	7.543
95% CI	2.575 to 3.636	5.000 to 5.000	6.000 to 6.000	7.317 to 7.769

q-CHA_2_DS_2_-VASc—quartile CHA_2_DS_2_-VASc score; N—number of patients; CI—confidence interval.

**Table 12:** The correlation between the quartiles of the mean values of the NLR and the CHA2DS2-VASc score for the surviving patients.

	NLR			
q-CHA_2_DS_2_-VASc	1	2	3	4
*n*	401	229	239	221
Minimum	0.0366	0.221	0.166	0.141
Maximum	38.875	25.800	28.333	28.579
Mean	4.117	5.242	5.465	5.151
95% CI	3.690 to 4.544	4.714 to 5.771	4.879 to 6.051	4.603 to 5.699

q-CHA_2_DS_2_-VASc—quartile CHA_2_DS_2_-VASc score; *n*—number of patients; CI—confidence interval.

## Data Availability

Data will be made available for valid written requests addressed to the corresponding author.
